# Disparities in Outpatient and Telehealth Visits During the COVID-19 Pandemic in a Large Integrated Health Care Organization: Retrospective Cohort Study

**DOI:** 10.2196/29959

**Published:** 2021-09-01

**Authors:** Lei Qian, Lina S Sy, Vennis Hong, Sungching C Glenn, Denison S Ryan, Kerresa Morrissette, Steven J Jacobsen, Stanley Xu

**Affiliations:** 1 Department of Research & Evaluation Kaiser Permanente Southern California Pasadena, CA United States

**Keywords:** COVID-19, pandemic, health care utilization, telehealth, disparity

## Abstract

**Background:**

Dramatic decreases in outpatient visits and sudden increases in telehealth visits were observed during the COVID-19 pandemic, but it was unclear whether these changes differed by patient demographics and socioeconomic status.

**Objective:**

This study aimed to assess the impact of the pandemic on in-person outpatient and telehealth visits (telephone and video) by demographic characteristics and household income in a diverse population.

**Methods:**

We calculated weekly rates of outpatient and telehealth visits by age, sex, race/ethnicity, and neighborhood-level median household income among members of Kaiser Permanente Southern California (KPSC) from January 5, 2020, to October 31, 2020, and the corresponding period in 2019. We estimated the percentage change in visit rates during the early pandemic period (March 22 to April 25, 2020) and the late pandemic period (October 4 to October 31, 2020) from the prepandemic period (January 5 to March 7, 2020) in Poisson regression models for each subgroup while adjusting for seasonality using 2019 data. We examined if the changes in visit rates differed by subgroups statistically by comparing their 95% CIs.

**Results:**

Among 4.56 million KPSC members enrolled in January 2020, 15.0% (n=682,947) were ≥65 years old, 51.5% (n=2,345,020) were female, 39.4% (n=1,795,994) were Hispanic, and 7.7% (n=350,721) lived in an area of median household income <US $40,000. Increases in telehealth visits during the pandemic varied across subgroups, while decreases in outpatient visits were similar, except by age. Among age groups, the ≥65 years population had the least increase in telehealth visits (236.6%, 95% CI 228.8%-244.5%), with 4.9 per one person-year during the early pandemic period versus 1.5 per one person-year during the prepandemic period. During the same periods, across racial/ethnic groups, Hispanic individuals had the largest increase in telehealth visits (295.5%, 95% CI 275.5%-316.5%). Across income levels, telehealth visits in the low-income group increased the most (313.5%, 95% CI 294.8%-333.1%). The rate of combined outpatient and telehealth visits in the Hispanic, non-Hispanic Black, and low-income groups returned to prepandemic levels by October 2020.

**Conclusions:**

The Hispanic group and low-income group had the largest percentage increase in telehealth utilization in response to the COVID-19 pandemic. The use of virtual care potentially mitigated the impact of the pandemic on health care utilization in these vulnerable populations.

## Introduction

The COVID-19 pandemic has impacted every aspect of life since it started in late December 2019, including health care delivery in the United States. After the declaration of a national state of emergency concerning the pandemic on March 13, 2020, the Centers for Disease Control and Prevention (CDC) and Centers for Medicare and Medicaid Services recommended delaying elective and nonemergent care on March 18, 2020 [1]. In addition, the CDC encouraged the use of telehealth services to deliver care [2]. Due to these policy changes and patients’ concerns about COVID-19 infection during health care visits, dramatic decreases in outpatient visits and sudden increases in telehealth visits (telephone and video) were observed during the COVID-19 pandemic [3-5].

However, the changes in outpatient and telehealth visits caused by the pandemic may differ by demographic and socioeconomic characteristics such as age, sex, race/ethnicity, language preference, income, and education [6,7]. Although telehealth visits increased prior to the pandemic primarily due to increased internet availability, insurance coverage, and cost savings [8], access to telehealth has inherent barriers [9,10]. Individuals with limited digital literacy or access to digital technology, limited health literacy, or limited English proficiency may not have the means to pursue telehealth technologies [11]. During the COVID-19 pandemic, these barriers may influence the existing disparities in accessing telehealth visits among individuals of low socioeconomic status, individuals of racial and ethnic minorities, and women [12].

Knowledge about utilization disparities during the pandemic is limited, and there remains great interest in understanding utilization disparities in large and diverse health care organizations. The objective of this study was to assess the impact of the COVID-19 pandemic on in-person outpatient and telehealth visits (telephone and video) in subgroups defined by age, sex, race/ethnicity, and neighborhood-level median household income in a large diverse population through October 31, 2020.

## Methods

### Study Setting, Population, and Period

This retrospective cohort study was conducted at Kaiser Permanente Southern California (KPSC), an integrated health care system that provides comprehensive health care to over 4.7 million racially, ethnically, and socioeconomically diverse members at its 15 hospitals and 234 medical offices [13]. The prepaid health plan provides strong motivation for members to use services at KPSC facilities. KPSC’s electronic health record system stores information about all aspects of the care provided to members, including demographics, medical encounters, vaccinations, pharmacy utilization, membership history, and claims. The study population consisted of individuals of all ages who were KPSC members from January 5, 2020, to October 31, 2020 (week 1 to week 43 in 2020) and the corresponding period in 2019.

### Outpatient and Telehealth Visits

We identified outpatient visits and telehealth visits using electronic health record data and claims data. The outpatient visits included all in-person outpatient visits, while telehealth visits included telephone appointment visits and video visits that were conducted synchronously using real-time telephone or live video-audio interaction. These telehealth visits were billable and had a diagnosis or procedure code. Less than 6% of outpatient and telehealth encounters in KPSC were from claims [14]. The study was reviewed and approved by the KPSC Institutional Review Board.

### Statistical Analysis

We first described the characteristics of KPSC members enrolled in January 2020. The weekly (Sunday to Saturday) rates of outpatient visits, telehealth visits, and combined outpatient and telehealth visits were calculated during the study period by age (0-5 years, 6-17 years, 18-44 years, 45-64 years, and ≥65 years), sex, self-reported race/ethnicity (non-Hispanic White, non-Hispanic Black, Hispanic, non-Hispanic Asian, and other/unknown), and neighborhood-level median household income (<US $40,000, US $40,000-$79,999, and ≥US $80,000). In calculating a weekly rate, the numerator was the visit count of each visit type and the denominator was person-years of membership during a given week. The trend of visit rates during the pandemic year by subgroups was plotted separately for outpatient, telehealth, and combined outpatient and telehealth visits.

To assess the impact of the COVID-19 pandemic on the visit rate, we selected the following three periods in 2020: week 1-9 (January 5 to March 7, 2020) for the prepandemic period, week 12-16 (March 22 to April 25, 2020) for the early pandemic period immediately after California’s stay-at-home order, and week 40-43 (October 4 to October 31, 2020) for the later pandemic period after reopening. Three corresponding periods in 2019 were also identified and used to control for seasonality. We estimated the ratio of the visit rate during the pandemic period to the visit rate during the prepandemic period in Poisson regression models with the visit count as the dependent variable and natural logarithm of person-years of membership as an offset for each subgroup, adjusting for other subgroup variables and seasonality. The robust variance estimator was used to obtain standard errors of parameter estimates in Poisson regression models. After the adjusted rate ratio was estimated from Poisson regression models, the percentage change was calculated as follows: (adjusted rate ratio − 1) × 100%. We examined if the changes in visit rates differed by age, sex, race/ethnicity, and household income by comparing their 95% CIs.

## Results 

### Study Population

Among the 4.56 million KPSC members enrolled in January 2020, 15.0% (n=682,947) were ≥65 years old, 51.5% (n=2,345,020) were female, 39.4% (n=1,795,994) were Hispanic, and 7.7% (n=350,721) lived in census tract areas with median household income <US $40,000 (Table 1).

**Table 1:** Demographic characteristics and neighborhood household income levels of members in Kaiser Permanente Southern California in January 2020 (N=4,556,646).

Characteristic		Value, n (%)
**Age (years)**		
	0-5	284,413 (6.2)
	6-17	653,649 (14.3)
	18-44	1,743,358 (38.3)
	45-64	1,192,279 (26.2)
	≥65	682,947 (15.0)
**Sex**		
	Female	2,345,020 (51.5)
	Male	2,211,304 (48.5)
	Unknown	322 (0.0)
**Race/ethnicity**		
	Hispanic	1,795,994 (39.4)
	Non-Hispanic White	1,403,840 (30.8)
	Non-Hispanic Black	347,019 (7.6)
	Non-Hispanic Asian	448,270 (9.8)
	Other and unknown	561,523 (12.3)
**Neighborhood median household income (US $)**		
	<40,000	350,721 (7.7)
	40,000-79,999	2,178,471 (47.8)
	≥80,000	1,913,377 (42.0)
	Unknown	114,077 (2.5)

### Outpatient Visits

**Figure 1:**
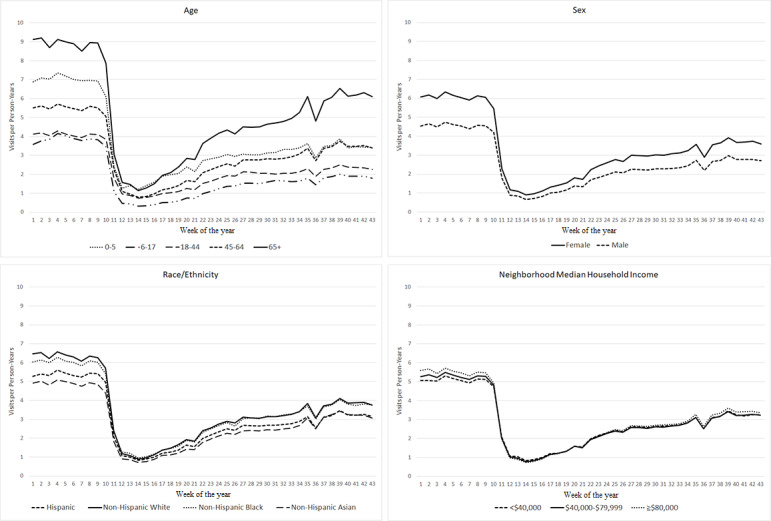
Outpatient visit rates over time by age, sex, race/ethnicity, and neighborhood median household income in 2020.

Across all subgroups, outpatient visit rates had a steep drop-off after the start of the pandemic, reaching their lowest levels by early April 2020 (week 14), and then, they gradually increased but remained lower than prepandemic levels (Figure 1). Among age groups, the ≥65 years population had the smallest decrease in outpatient visits (adjusted percentage change: −29.6%, 95% CI −30.5% to −28.6%), with 6.25 visits per person-year during the later pandemic period versus 8.92 visits per person-year during the prepandemic period in 2020 (Table 2). Children aged 6-17 years had the largest decrease (−50.2%, 95% CI −51.6% to −48.7%) in outpatient visits, with 1.92 visits per person-year during the later pandemic period versus 3.86 visits per person-year during the prepandemic period in 2020. The decreases in outpatient visits did not differ statistically by sex, race/ethnicity, and household income groups, ranging from −83.8% to −80.7% when comparing the early pandemic period to the prepandemic period in 2020 and ranging from −38.8% to −35.9% when comparing the later pandemic period to the prepandemic period in 2020.

**Table 2:** Outpatient visit rates per one person-year before and during the COVID-19 pandemic in 2020.

Characteristic		Prepandemic	During the pandemic		Percentage change from prepandemic (%)		Adjusted percentage change from prepandemic (%) (95% CI)^a^	
		Week 1-9	Week 12-16 (early)	Week 40-43 (later)	Week 12-16 (early)	Week 40-43 (later)	Week 12-16 (early)	Week 40-43 (later)
**Age (years)**								
	0-5	7.03	1.35	3.53	−80.8	−49.7	−79.6 (−80.7 to −78.5)	−47.2 (−48.9 to −45.4)
	6-17	3.86	0.38	1.92	−90.2	−50.3	−90.0 (−90.4 to −89.5)	−50.2 (−51.6 to −48.7)
	18-44	4.10	0.83	2.39	−79.6	−41.7	−78.9 (−79.5 to −78.3)	−40.8 (−42.0 to −39.4)
	45-64	5.52	0.93	3.52	−83.2	−36.2	−82.6 (−83.0 to −82.2)	−35.4 (−36.5 to −34.2)
	≥65	8.92	1.39	6.25	−84.4	−29.9	−84.1 (−84.5 to −83.8)	−29.6 (−30.5 to −28.6)
**Sex**								
	Female	6.08	1.03	3.73	−83.0	−38.7	−82.5 (−82.9 to −82.1)	−38.3 (−39.2 to −37.4)
	Male	4.55	0.78	2.80	−83.0	−38.4	−82.3 (−82.7 to −81.9)	−37.4 (−38.5 to −36.2)
**Race/ethnicity**								
	Hispanic	5.37	0.95	3.26	−82.2	−39.3	−81.7 (−82.8 to −80.5)	−38.8 (−41.6 to −35.8)
	Non-Hispanic White	6.34	1.03	3.90	−83.7	−38.4	−83.1 (−83.9 to −82.2)	−38.0 (−40.3 to −35.7)
	Non-Hispanic Black	6.04	1.11	3.82	−81.6	−36.8	−81.1 (−82.2 to −80.0)	−36.3 (−39.0 to −33.4)
	Non-Hispanic Asian	4.90	0.82	3.23	−83.2	−34.0	−83.0 (−84.0 to −81.9)	−35.9 (−38.5 to −33.1)
**Neighborhood median household income (US $)**								
	<40,000	5.09	0.97	3.23	−81.0	−36.5	−80.7 (−81.7 to −79.5)	−36.8 (−39.6 to −33.8)
	40,000-79,999	5.28	0.92	3.23	−82.7	−38.9	−82.2 (−83.2 to −81.2)	−38.7 (−41.2 to −36.1)
	≥80,000	5.51	0.87	3.40	−84.3	−38.3	−83.8 (−84.6 to −82.9)	−38.1 (−40.3 to −35.7)

^a^Analyses for age groups were adjusted for sex and race/ethnicity; analyses for sex were adjusted for age and race/ethnicity; analyses for race/ethnicity were adjusted for age and sex; analyses for neighborhood median household income were adjusted for age, sex, and race/ethnicity; all analyses were adjusted for seasonality using 2019 data.

### Telehealth Visits

**Figure 2:**
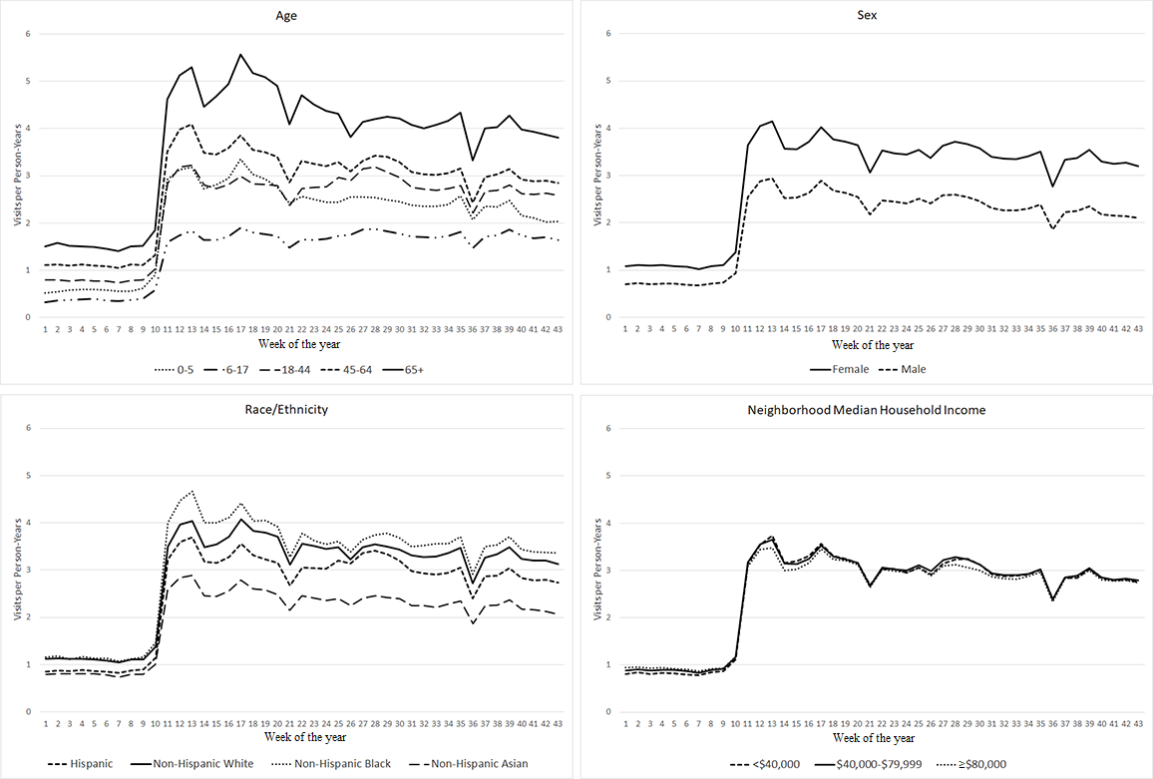
Telehealth visit rates over time by age, sex, race/ethnicity, and neighborhood median household income in 2020.

Across all subgroups, except children aged 6-17 years, telehealth visit rates increased sharply after the start of the pandemic and then slowly decreased (Figure 2). The telehealth visit rates in children aged 6-17 years remained stable after an initial increase during the pandemic. Comparing the early pandemic period to the prepandemic period in 2020, across age groups, the telehealth visits increased the most among children aged 0-5 years (475.5%, 95% CI 446.2%-506.4%) and the least among adults aged ≥65 years (236.6%, 95% CI 228.8%-244.5%) (Table 3). Telehealth visits increased significantly more among males (293.9%, 95% CI 283.4%-304.7%) than females (260.0%, 95% CI 252.6%-267.6%). Across racial/ethnic groups, Hispanic individuals had the largest increase in telehealth visits (295.5%, 95% CI 275.5%-316.5%). Across income levels, telehealth visits in the low-income group increased the most (313.5%, 95% CI 294.8%-333.1%). The patterns were similar when comparing the later pandemic period to the prepandemic period in 2020, but with smaller differences.

**Table 3:** Telehealth visit rates per one person-year before and during the COVID-19 pandemic in 2020.

Characteristic		Prepandemic	During the pandemic		Percentage change from prepandemic (%)		Adjusted percentage change from prepandemic (%) (95% CI)^a^	
		Week 1-9	Week 12-16 (early)	Week 40-43 (later)	Week 12-16 (early)	Week 40-43 (later)	Week 12-16 (early)	Week 40-43 (later)
**Age (years)**								
	0-5	0.57	2.97	2.24	422.2	293.4	475.5 (446.2-506.4)	354.2 (331.4-378.2)
	6-17	0.37	1.72	1.77	370.4	383.6	383.5 (362.9-405.1)	394.8 (372.9-417.7)
	18-44	0.78	2.95	2.65	278.5	239.6	282.6 (271.2-294.3)	237.6 (228.3-247.2)
	45-64	1.10	3.72	2.91	238.5	165.2	247.7 (237.8-257.8)	164.0 (157.0-171.2)
	≥65	1.49	4.90	3.91	227.7	161.5	236.6 (228.8-244.5)	161.1 (156.2-166.2)
**Sex**								
	Female	1.08	3.81	3.30	252.2	204.9	260.0 (252.6-267.6)	204.0 (197.1-211.0)
	Male	0.70	2.70	2.19	283.7	211.0	293.9 (283.4-304.7)	212.4 (203.1-222.0)
**Race/ethnicity**								
	Hispanic	0.86	3.37	2.83	291.3	227.6	295.5 (275.5-316.5)	220.3 (203.0-238.5)
	Non-Hispanic White	1.10	3.75	3.23	240.4	193.3	255.2 (240.7-270.3)	198.9 (186.0-212.4)
	Non-Hispanic Black	1.13	4.25	3.43	274.1	202.5	278.6 (260.1-298.0)	188.3 (173.7-203.6)
	Non-Hispanic Asian	0.79	2.63	2.18	233.7	176.1	233.2 (215.7-251.7)	169.0 (153.7-185.3)
**Neighborhood median household income (US $)**								
	<40,000	0.82	3.38	2.79	311.7	239.5	313.5 (294.8-333.1)	220.4 (204.7-236.9)
	40,000-79,999	0.88	3.34	2.81	277.6	217.8	283.3 (267.5-299.7)	211.4 (197.6-225.8)
	≥80,000	0.92	3.22	2.77	249.0	200.7	259.3 (245.4-273.8)	202.4 (189.9-215.5)

^a^Analyses for age groups were adjusted for sex and race/ethnicity; analyses for sex were adjusted for age and race/ethnicity; analyses for race/ethnicity were adjusted for age and sex; analyses for neighborhood median household income were adjusted for age, sex, and race/ethnicity; all analyses were adjusted for seasonality using 2019 data.

### Combined Outpatient and Telehealth Visits

**Figure 3:**
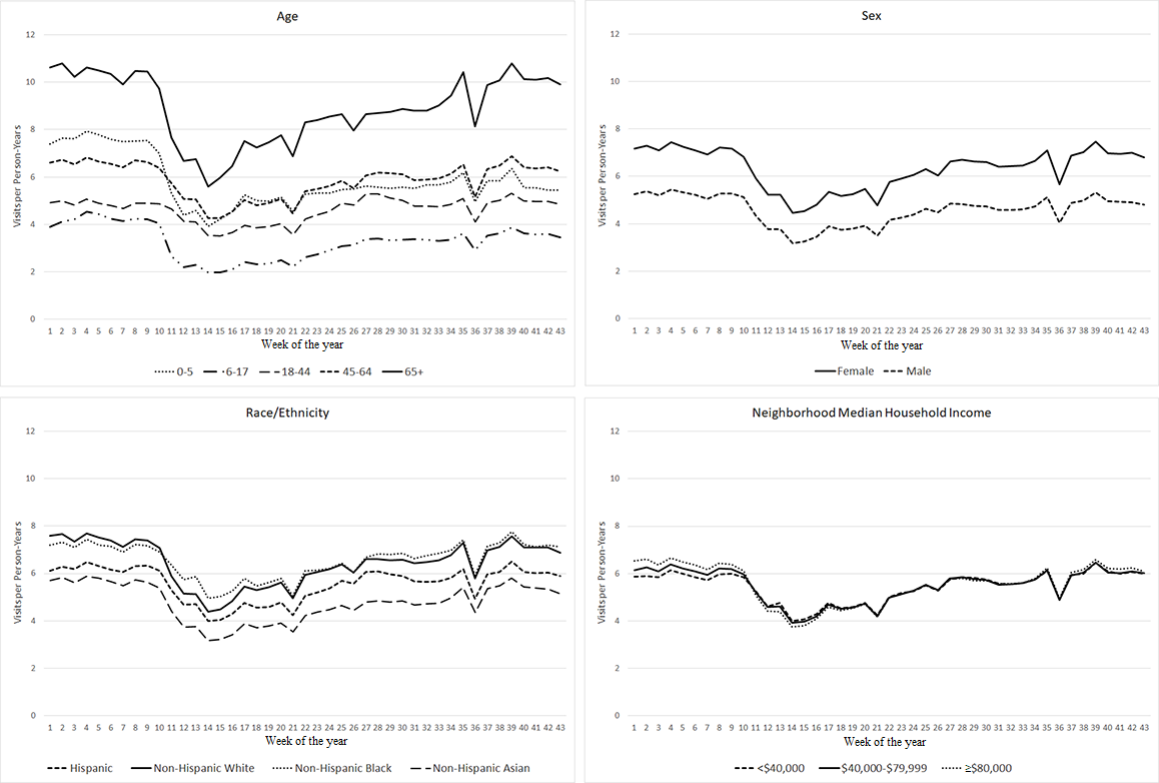
Combined outpatient and telehealth visit rates over time by age, sex, race/ethnicity, and neighborhood median household income in 2020.

Despite increases in telehealth visits during the pandemic, across all subgroups, the rate of combined telehealth and outpatient visits still decreased during the early pandemic period and only returned to prepandemic levels for some subgroups (Figure 3). Comparing the later pandemic period to the prepandemic period in 2020, across age groups, the rate of combined outpatient and telehealth visits decreased the most among children aged 0-5 years (−19.7%, 95% CI −21.9% to −17.5%) (Table 4). The combined visit rate among adults aged 18-44 years during the later pandemic period exceeded the prepandemic level by 4.7% (95% CI 2.5%-6.8%). The combined visit rates returned to the prepandemic levels by October 2020 for the following subgroups: Hispanics (percentage change: −1.8%, 95% CI −6.1% to 2.6%), Non-Hispanic Black individuals (percentage change: 1.1%, 95% CI −3.0% to 5.3%), those with income <US $40,000 (percentage change: 0.7%, 95% CI −3.4% to 5.0%), and those with income US $40,000-$79,999 (percentage change: −2.1%, 95% CI −5.8% to 1.8%).

**Table 4:** Combined outpatient and telehealth visit rates per one person-year before and during the COVID-19 pandemic in 2020.

Characteristic		Prepandemic	During the pandemic		Percentage change from prepandemic (%)		Adjusted percentage change from prepandemic (%) (95% CI)^a^	
		Week 1-9	Week 12-16 (early)	Week 40-43 (later)	Week 12-16 (early)	Week 40-43 (later)	Week 12-16 (early)	Week 40-43 (later)
**Age (years)**								
	0-5	7.60	4.32	5.77	−43.1	−24.0	−39.5 (−41.5 to −37.4)	−19.7 (−21.9 to −17.5)
	6-17	4.22	2.10	3.69	−50.2	−12.7	−49.2 (−50.6 to −47.7)	−12.3 (−14.6 to −9.9)
	18-44	4.88	3.79	5.04	−22.3	3.3	−19.8 (−21.4 to −18.2)	4.7 (2.5 to 6.8)
	45-64	6.62	4.64	6.43	−29.9	−2.8	−27.4 (−28.7 to −26.0)	−1.8 (−3.4 to −0.1)
	≥65	10.41	6.28	10.16	−39.6	−2.5	−38.5 (−39.4 to −37.5)	−2.1 (−3.3 to −0.9)
**Sex**								
	Female	7.16	4.84	7.02	−32.4	−2.0	−30.6 (−31.7 to −29.5)	−1.4 (−2.7 to −0.1)
	Male	5.26	3.48	4.99	−33.9	−5.0	−31.4 (−32.6 to −30.2)	−3.5 (−5.1 to −2.0)
**Race/ethnicity**								
	Hispanic	6.23	4.33	6.08	−30.5	−2.4	−28.7 (−31.8 to −25.5)	−1.8 (−6.1 to 2.6)
	Non-Hispanic White	7.44	4.78	7.13	−35.8	−4.1	−33.3 (−35.6 to −30.9)	−3.4 (−6.6 to 0.0)
	Non-Hispanic Black	7.18	5.36	7.25	−25.3	1.0	−23.5 (−26.7 to −20.3)	1.1 (−3.0 to 5.3)
	Non-Hispanic Asian	5.69	3.45	5.41	−39.3	−4.8	−38.4 (−41.0 to −35.7)	−7.5 (−11.1 to −3.7)
**Neighborhood median household income (US $)**								
	<40,000	5.91	4.35	6.02	−26.5	1.9	−25.2 (−28.3 to −22.1)	0.7 (−3.4 to 5.0)
	40,000-79,999	6.17	4.26	6.04	−31.0	−2.1	−29.2 (−31.9 to −26.4)	−2.1 (−5.8 to 1.8)
	≥80,000	6.43	4.08	6.17	−36.5	−4.1	−34.4 (−36.7 to −32.1)	−3.7 (−6.9 to −0.3)

^a^Analyses for age groups were adjusted for sex and race/ethnicity; analyses for sex were adjusted for age and race/ethnicity; analyses for race/ethnicity were adjusted for age and sex; analyses for neighborhood median household income were adjusted for age, sex, and race/ethnicity; all analyses were adjusted for seasonality using 2019 data.

## Discussion

### Principal Findings

We examined the impact of the COVID-19 pandemic on outpatient and telehealth visits by demographic characteristics and household income levels in a large integrated health care system. We found that increases in telehealth visits during the COVID-19 pandemic varied across age, sex, race/ethnicity, and household income groups, while decreases in outpatient visits during the COVID-19 pandemic were similar across all subgroups, except age groups. The rates of combined outpatient and telehealth visits in the Hispanic, non-Hispanic Black, and low-income groups returned to prepandemic levels by October 2020.

While adults aged ≥65 years had the highest telehealth visit rates before and during the pandemic, this age group had the smallest percentage increase in telehealth visits during the pandemic among all age groups, suggesting that greater barriers existed for the senior population to further expand their use of telehealth services during the pandemic. For example, seniors may have limited access to digital technologies and may have difficulty in using technologies required for telehealth visits. Recent studies among Medicare beneficiaries showed that 26.3% of individuals lacked digital access at home, making it unlikely for them to have video visits with clinicians [15]. Furthermore, a recent study showed that difficulty hearing and problems of speaking or making oneself understood were the top two reasons for hesitancy to use telehealth [16].

Members living in an area with median household income <US $40,000 increased their telehealth visits per person-year from 0.82 during week 1-9 to 3.38 during week 12-16, a higher percentage increase than that for those living in an area with median household income ≥US $80,000 (from 0.92 during week 1-9 to 3.22 during week 12-16). The KPSC service area covers mostly nonrural residents whose access to a telephone is not a barrier for using telehealth services. Following the CDC’s recommendation to delay elective and nonemergent care on March 18, 2020, KPSC offered zero co-payments for telehealth visits, which might have been especially helpful for the low-income population. In addition, telehealth visits provide flexibility in care location, and save time and cost associated with commuting for in-person care.

Hispanic individuals had the greatest percentage increase in telehealth visits across racial/ethnic groups, although their visit rates during the prepandemic and pandemic periods were lower than those of non-Hispanic Black and non-Hispanic White individuals. A study conducted in New York City during the pandemic showed that Hispanic and Black patients had lower odds of using telehealth visits versus office visits than either White or Asian patients, but the authors did not consider the trend of telehealth utilization [17]. A recent study based on survey data showed that Black respondents were more likely than White respondents to report using telehealth because of the pandemic [18]. The fact that a larger proportion of racial/ethnic minorities live in low-income areas, along with KPSC’s zero co-payment policy for telehealth visits, might support telehealth care among Hispanic individuals. In addition, telehealth is accessible for members with limited English proficiency. Spanish is a common language in southern California, spoken by many providers. The removal of Spanish language barriers could have encouraged Hispanic individuals to continue using telehealth care and contributed to larger increases of telehealth care in this population.

The impact of the COVID-19 pandemic on telephone and video visits may differ because the barrier to technology use is higher for video than telephone [12]. We assessed the rate of telehealth visits within KPSC separately by telephone and video visits (Multimedia Appendix 1). The trend of telephone visits was similar to the trend of overall telehealth visits, with the rates increasing sharply after the start of the pandemic and then gradually decreasing. The use of video visits was very low before the pandemic, with only 2.1% of telehealth visits occurring within KPSC being video visits. The video visit rate increased gradually over time after the start of the pandemic, contributing to 24% of telehealth visits by the end of October 2020. The slower and more gradual increase in video visits was likely due to technology barriers, for example, need for a web camera and digital literacy to conduct a video visit. These barriers were more prominent in those aged ≥65 years (Multimedia Appendix 2), with video visits having a slower start compared to that in some other age groups. In addition, it took time for both members and providers to adopt this new approach, with gradual increases in video visits occurring across all subgroups. Given the growing familiarity with new telehealth technologies, even as the COVID-19 pandemic wanes, patients likely will continue to pursue telehealth services beyond pre-COVID-19 levels.

### Limitations

There are some potential limitations in this study. First, while we studied the impact of the pandemic on health care utilization among subpopulations, we did not address the quality of care and population health. Second, we assessed members’ neighborhood-level income based on their address, which is less accurate than individual-level income. Third, because KPSC’s health plan, hospitals, and medical groups are integrated to create a system for coordinated and comprehensive patient care, our patients may have better access to care than the general population. Therefore, these results may not be generalizable to populations in less integrated health care settings. Finally, we focused on relative change (ie, percentage change from baseline) in utilization as part of this study, but we acknowledge that absolute change (ie, difference from baseline) in utilization may be of interest as well. As such, we have presented absolute visit rates in each time period by subgroup to allow for such comparisons to be made.

### Conclusion

While the ≥65 years population had the least percentage increase in telehealth visits, the Hispanic and low-income groups had the greatest percentage increase in telehealth utilization, possibly due to the low cost and flexibility of location and time. The use of virtual care in these subgroups reduced exposure to COVID-19, reinforced social distancing protocols, increased the number of patients who could be treated, and potentially mitigated the impact of COVID-19 in vulnerable populations. Furthermore, the use of virtual care might be a promising way to reduce health care disparities even after the COVID-19 pandemic ends.

## Appendix

Multimedia Appendix 1 Telephone and video visit rates within Kaiser Permanente Southern California over time in 2020.

Multimedia Appendix 2 Video visit rates within Kaiser Permanente Southern California over time by age, sex, race/ethnicity, and neighborhood median household income in 2020.

## Abbreviations

CDC: Centers for Disease Control and Prevention
KPSC: Kaiser Permanente Southern California
