# Supplementary figures and images for: Disparities in Outpatient and Telehealth Visits During the COVID-19 Pandemic in a Large Integrated Health Care Organization: Retrospective Cohort Study
Source: J Med Internet Res. 2021 Sep 1;23(9):e29959. doi: 10.2196/29959 (PMC8412134; doi:10.2196/29959)

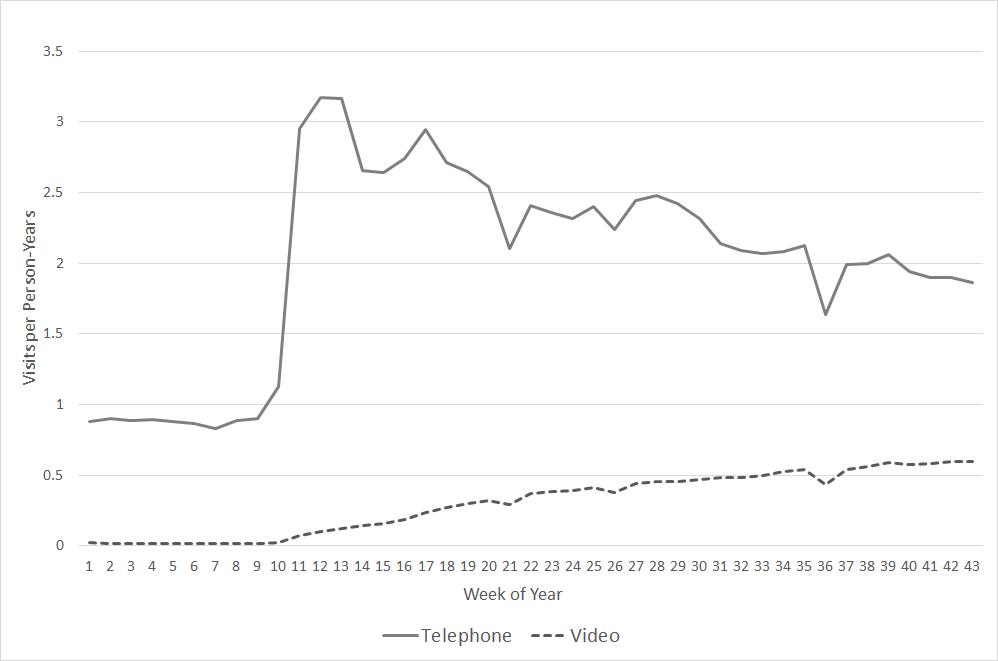

Supplement: Multimedia Appendix 1 [file jmir_v23i9e29959_app1.png]

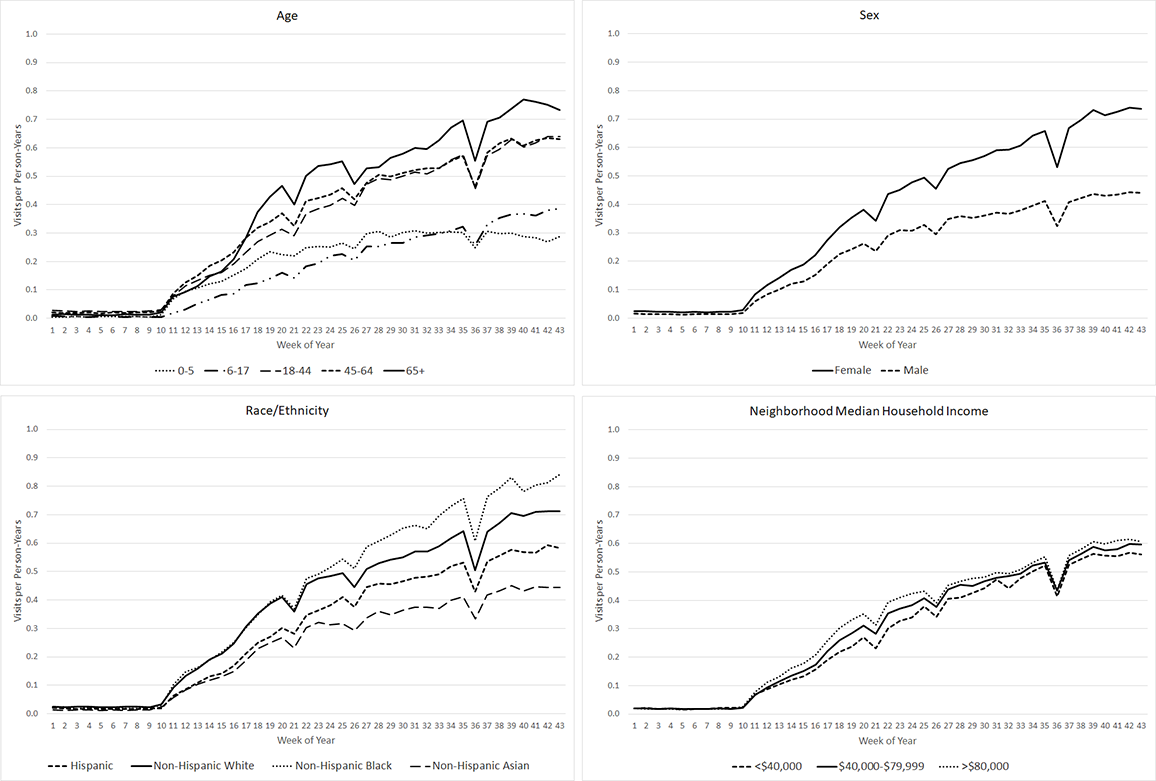

Supplement: Multimedia Appendix 2 [file jmir_v23i9e29959_app2.png]
